# Prevalence and antimicrobial resistance of *Salmonella* spp. isolated from swine and poultry farms in Cambodia

**DOI:** 10.14202/vetworld.2025.918-926

**Published:** 2025-04-23

**Authors:** Bunna Chea, Sokom Kong, Sokha Thim, Naiheak Ban, Rithy Chrun, Vutey Venn, Cherry Fernandez-Colorado, Kroesna Kang

**Affiliations:** 1 Faculty of Veterinary Medicine, Royal University of Agriculture, Phnom Penh, Cambodia; 2 Faculty of Agro-Industry, Royal University of Agriculture, Phnom Penh, Cambodia; 3 National Animal Health and Production Research Institute, General Directorate of Animal Health and Production, Phnom Penh, Cambodia; 4 Department of Veterinary Paraclinical Sciences, College of Veterinary Medicine, University of the Philippines Los Baños, College, Los Baños, Laguna, Philippines

**Keywords:** antimicrobial resistance, Cambodia, multidrug resistance, poultry, *Salmonella*, swine

## Abstract

**Background and Aim:**

The indiscriminate use of antimicrobials in livestock farming has contributed to the emergence of antimicrobial-resistant pathogens, posing a significant public health challenge. This study aimed to assess the prevalence and antimicrobial resistance (AMR) profiles of *Salmonella* spp. isolated from swine and poultry in small- and medium-scale farms in Cambodia.

**Materials and Methods:**

A total of 638 fecal samples (273 rectal swabs from swine and 365 cloacal swabs from poultry) were collected from five provinces in Cambodia from June to September 2021. *Salmonella* spp. isolation followed ISO 6579:2002 guidelines, and antimicrobial susceptibility testing was conducted using the disk diffusion method, adhering to Clinical and Laboratory Standards Institute 2020 standards. Resistance was assessed against nine antimicrobial agents across five major classes.

**Results:**

Overall, *Salmonella* was detected in 6.58% (42/638) of samples, with 6.96% (19/273) from swine and 6.30% (23/365) from poultry. Swine-derived isolates exhibited the highest prevalence in the growing stage (13.93%), whereas poultry isolates were most common in avian broilers (14.55%). High resistance was observed against β-lactams (penicillin, amoxicillin, and ampicillin), tetracyclines, and sulfonamides, with resistance rates ranging from 73.81% to 100%. Fluoroquinolone resistance (ciprofloxacin and norfloxacin) was moderate (29.77%), while aminoglycoside resistance (gentamicin) was low (2.38%). Multidrug resistance (MDR) patterns were identified in 91.30% (21/23) of poultry isolates and 52.63% (10/19) of swine isolates, with resistance spanning three to five antimicrobial classes.

**Conclusion:**

The presence of *Salmonella* in Cambodian swine and poultry farms and its high level of MDR underscore the urgent need for improved antimicrobial stewardship. The study highlights the risk of MDR *Salmonella* transmission through livestock production chains, emphasizing the necessity for stringent regulatory interventions, biosecurity measures, and surveillance programs to mitigate AMR spread in animal agriculture and public health sectors.

## INTRODUCTION

*Salmonella* is a major foodborne zoonotic patho-gen with significant global public health implications. It is responsible for approximately 78 million enteric infections and 59,153 diarrheal deaths annually [1]. Human salmonellosis is primarily associated with the consumption of contaminated food products such as pork, poultry meat, eggs, and other meat products [1–4]. In addition, direct contact with infected animals, including chickens, swine, and cattle, has been identified as a transmission route [5–8].

The emergence of antimicrobial resistance (AMR) in *Salmonella* presents a growing challenge to public health, food security, and global economic development. Without effective intervention, AMR could lead to millions of deaths annually, with estimates predicting up to 10 million fatalities by 2050 [9, 10]. In the livestock sector, AMR in *Salmonella* is particularly concerning due to the widespread use of antimicrobials for disease treatment, prophylaxis, and growth promotion [11]. The increasing demand for animal products is expected to drive a twofold increase in antimicrobial use (AMU) in swine and poultry production [12]. Consequently, excessive antimicrobial exposure facilitates the sele-ction and dissemination of resistant *Salmonella* strains, threatening both animal and human health [13].

In Cambodia, the livestock industry plays a critical role in food security and economic stability. In 2022, the country had 606 commercial swine farms and 730 poultry farms, producing 2,210,996 pigs and 24,240,290 poultry [14]. Given the scale of livestock production, monitoring *Salmonella* prevalence and AMR trends is crucial for safeguarding public health. Several studies by Tadee *et al*. [15], Tu *et al*. [16], Lettini *et al*. [17], Huynh and Ly [18], Anuchatkitcharoen *et al*. [19] and Prasertsee *et al*. [20] in Thailand and Vietnam have reported *Salmonella* prevalence rates ranging from 14.58% to 87% in swine and poultry farms, with AMR levels as high as 100% against multiple antimicrobial classes. In particular, *Salmonella* isolates from Vietnam and Thailand have demonstrated frequent resistance to tetracycline (TET), ciprofloxacin (CIP), ampicillin (AMP), and sulfamethoxazole, with multidrug resistance (MDR) patterns spanning multiple antimicrobial classes in both poultry and swine setting [3, 17, 18, 21, 22].

Despite growing concerns over AMR in *Salmonella*, there are limited data on the prevalence and AMR profiles of *Salmonella* in Cambodian commercial swine and poultry farms. While studies by Trongjit *et al*. [8] and Lay *et al*. [23] have identified MDR *Salmonella* in slaughtered swine, poultry, and their products in border provinces and Phnom Penh, systematic surveillance at the farm level remains inadequate. In addition, *Escherichia coli* strains exhibiting MDR have been isolated from live swine and poultry [24, 25]. Furthermore, high AMR levels have been reported among *Campylobacter* spp. isolates from raw poultry sold at retail markets in Phnom Penh [23]. Moreover, previous studies have primarily focused on post-slaughter contamination rather than on-farm prevalence and resistance patterns. The absence of baseline data hinders the development of targeted interventions to mitigate the risks of AMR in livestock production systems.

This study aimed to fill this knowledge gap by determining the prevalence and AMR profiles of *Salmonella* isolates from small- and medium-scale commercial swine and poultry farms in Cambodia. By identifying specific AMR patterns and MDR prevalence in *Salmonell*a isolates, this study provides evidence-based insights to inform disease control strategies, antimicrobial stewardship programs, and policy development to reduce the burden of foodborne salmonellosis in Cambodia.

## MATERIALS AND METHODS

### Ethical approval

This study was reviewed and approved by the Faculty of Veterinary Medicine, Royal University of Agriculture.

### Study period and location

The study was conducted from June to September 2021 across five provinces in Cambodia - Kampong Speu, Svay Rieng, Takeo, Kandal, and Prey Veng.

### Sample collection

This study was a continuation of a previous investigation that assessed the knowledge, attitudes, and practices (KAP) of swine and poultry farms [26]. A total of 638 samples were aseptically collected as per the sample size determined in the previous study [26], consisting of 273 rectal swabs from swine and 365 cloacal swabs from poultry, sourced from small- and medium-scale commercial farms (Table 1).

**Table 1:** Number of farms and samples collected by production type for swine and poultry.

Species	Farm ID	Farm scale*	Production type	No. of samples
Swine	A	Medium	Weaning, growing, and gilts	45
	B	Medium	Growing	73
	C	Medium	Finishing	56
	D	Medium	Finishing	40
	E	Medium	Weaning and growing	30
	F	Small	Weaning and growing	29
Subtotal	6			273
Poultry	A	Small	Broiler (Avian breed)	55
	B	Small	Broiler (Three blood breed)	50
	C	Small	Broiler (Three blood breed)	64
	D	Small	Broiler (Three blood breed)	57
	E	Medium	Broiler (Three blood breed)	35
	F	Medium	Broiler (Three blood breed)	35
	G	Small	Broiler (Local chicken breed)	39
	H	Small	Layer duck	30
Subtotal	8			365
Total	14			638

Swine: Small-scale (Fattening swine 100 - <1000 heads; breeders 50 - <200 heads); medium-scale (Fattening swine 1000 - <5000 heads; breeders 200 - <500 heads). Poultry: Small-scale (Broiler 5000 -<30000 heads; ducks 5000 - <20000 heads); Medium-scale (Broiler 30000 -<50000 heads; ducks 20000 - <50000 heads)

Commercial swine and poultry farms were purposefully selected from each province for sample collection. Swine farms were sampled based on produ-ction stages, including weaning, growing, finishing, and gilts. Due to restrictions imposed by the African Swine Fever outbreak, only six swine farms were visited during the sampling period. Among these, samples were collected from three farms with multiple production stages, while the remaining three farms were sampled at a single production stage. Poultry samples were collected from eight farms representing various production types, including layer ducks and broiler chickens (avian, three-blood, and local breeds). A minimum of 24 samples per farm or production type were collected from animals exhibiting clinical signs of diarrhea, following sample size estimations for prevalence in a large population [27]. If the required number of diarrheic animals was not met, additional individuals from the affected group were included.

All samples were collected from each farm during a single sampling event. Specimens were transported in ice containers to the laboratory at the Faculty of Veterinary Medicine, Royal University of Agriculture, Phnom Penh, Cambodia. Upon arrival, samples were immediately placed in peptone water on the same day and cultured the following day for further analysis.

### Isolation and identification of *Salmonella*

Samples were collected, and bacterial culture was performed following the standard industry procedure [28]. Briefly, individual swabs were immersed in 10 mL of buffered peptone water (BPW) (Merck, Germany) and incubated for 18–22 h at 37°C. Subsequently, 0.1 mL of the incubated BPW was transferred into 9 mL of Rappaport-Vassiliadis medium (Merck, Germany) and incubated for an additional 22–24 h at 37°C. The isolates were then sub-cultured on xylose lysine deoxycholate (XLD) agar plates (Merck, Germany) and incubated for 22–24 h at 37°C.

From each sample, 2–3 suspected colonies on XLD agar were selected for biochemical confirmation using a combination of tests, including Triple Sugar Iron, Catalase test, Motility-Indole-Lysine test, and API 20E.

### AMR testing of *Salmonella*

Antimicrobial susceptibility testing was conducted using the disk diffusion method in accordance with the Clinical and Laboratory Standards Institute (CLSI) guidelines [29]. Initially, 2–3 *Salmonella* colonies grown overnight on Nutrient Agar were suspended in 5 mL of 0.9% sterile saline (NaCl) and adjusted to match the 0.5 McFarland standard turbidity. A sterile cotton swab was dipped into the suspension and evenly spread over Mueller-Hinton Agar (Merck, Germany). Antimicrobial disks were then placed on the agar surface, and the plates were incubated for 16–18 h at 37°C.

Following incubation, the diameters of the inhibition zones surrounding the antimicrobial disks were measured and interpreted according to CLSI guidelines [29]. The antimicrobial agents tested included penicillin (PG) 10 U, amoxicillin (AML) 30 µg, AMP 10 µg, amoxicillin-clavulanate acid (AMC) 30 µg, CIP 5 µg, norfloxacin (NOR) 10 µg, TET 30 µg, gentamicin (GEN) 10 µg, and sulfamethoxazole-trimethoprim (SXT) 25 µg. *Salmonella* isolates were classified as MDR if they exhibited resistance to at least one antimicrobial agent in three or more antimicrobial classes.

### Statistical analysis

Prevalence and AMR patterns were analyzed using descriptive statistics. Chi-square tests were performed to determine significant differences in *Salmonella* prevalence across animal production types and MDR patterns. A p-value < 0.05 was considered statistically significant. Data analysis was conducted using the Statistical Package for the Social Sciences software, version 16 (IBM Corp., NY, USA).

## RESULTS

### *Salmonella* prevalence and resistance

The prevalence of *Salmonella* spp. varied according to the production type within each species (Table 2). The overall detection rate of *Salmonella* spp. in diarrheic samples was 6.58% (42/638), with a frequency of 6.96% (19/273) in swine and 6.30% (23/365) in poultry. Among the swine production stages, growing swine exhibited the highest prevalence (13.93%), which was significantly greater than that observed in weaning (2.56%) and finishing swine (1.04%) (p < 0.001). No *Salmonella* species were detected in gilts. In poultry, the frequencies of *Salmonella* spp. did not differ significantly (p > 0.05) among avian broilers (14.55%), layer ducks (6.67%), three-blood broilers (4.56%), and local chickens (5.13%).

**Table 2:** Prevalence of *Salmonella* spp. according to production type in swine and poultry.

Species	Production type	No. of farms	No. of samples	No. of positive samples, n (%)	p-value
Swine	Weaning	3*	39	1 (2.56)	0.001
	Growing	4*	122	17 (13.93)	
	Finishing	2	96	1 (1.04)	
	Gilts	1*	16	0 (0.00)	
	Subtotal	6	273	19 (6.96)	
Poultry	Broiler (Avian breed)	1	55	8 (14.55)	0.054
	Broiler (Three blood breed)	5	241	11 (4.56)	
	Broiler (Local chicken breed)	1	39	2 (5.13)	
	Layer duck	1	30	2 (6.67)	
	Subtotal	8	365	23 (6.30)	
	Total	14	638	42 (6.58)	

* Two farms were weaning and growing and one farm were mixed weaning, growing, and gilts

Overall, of the *Salmonella* isolates that were resistant to fluoroquinolones, the level of resistance was only moderate but was high for those resistant to β-lactams, tetracyclines, and sulfonamides. In contrast, aminoglycosides were associated with low resistance among the isolates (Table 3). Among β-lactams antibiotics, AMC acid was the only agent with low resistance. A *Salmonella* isolate from swine exhibited high resistance to both β-lactams and tetracyclines. Fluoroquinolones displayed low or negligible resistance, whereas sulfonamides exhibited moderate resistance. In poultry-derived *Salmonella* isolates, resistance was notably high against tetracyclines, sulfonamides, and β-lactams, whereas fluoroquinolones demonstrated moderate resistance. However, aminoglycosides sho-wed minimal resistance.

**Table 3:** Antimicrobial resistance profiles of *Salmonella* spp. in swine and poultry swab samples.

Antimicrobial classes	Antimicrobial agents	Number of *Salmonella* spp. isolates that are resistant		

		Swine (n = 19)	Poultry (n = 23)	Total (n = 42)
β-lactams	P (G)	19 (100)	23 (100)	42 (100)
	AML	19 (100)	16 (69.57)	35 (83.33)
	AMP	19 (100)	14 (60.87)	33 (78.57)
	AMC	0 (0.00)	1 (4.35)	1 (2.38)
Fluoroquinolones	CIP	2 (10.53)	15 (65.22)	17 (40.48)
	NOR	0 (0.00)	8 (34.78)	8 (19.05)
Tetracyclines	TET	19 (100)	23 (100)	42 (100)
Sulfonamides	SXT	10 (52.63)	21 (91.30)	31 (73.81)
Aminoglycosides	GEN	0 (0.00)	1 (4.35)	1 (2.38)

P (G)=Penicillin G, AML=Amoxicillin, AMP=Ampicillin, AMC=Amoxicillin-clavulanate acid, CIP=Ciprofloxacin, NOR=Norfloxacin, TET=Tetracycline, GEN=Gentamicin, SXT=Sulfamethoxazole-trimethoprim

Nine distinct MDR patterns were identified among the 42 *Salmonella*-positive isolates (Table 4). The majority of *Salmonella* isolates from poultry, 21/23 (91.30%) exhibited resistance to three to five antimicrobial classes, whereas in swine, 10/19 (52.63%) isolates displayed MDR to three to four antimicrobial classes (p < 0.001). Among the poultry isolates, 14/23 (60.87%) were resistant to four antimicrobial classes, whereas 6/23 (25.08%) exhibited resistance to three classes. Only 1/23 (4.35%) demonstrated resistance to the five antimicrobial classes. In swine isolates, the most frequently observed MDR pattern involved resistance to three antimicrobial classes, namely, tetracyclines, sulfonamides, and β-lactams, detected in 8/19 (42.10%) isolates. In addition, 2/19 (10.53%) swine isolates exhibited resistance to four antimicrobial classes: tetracyclines, sulfonamides, β-lactams, and fluoroquinolones.

**Table 4:** Multidrug resistance patterns of *Salmonella* spp. in swine and poultry.

Resistance pattern	Antimicrobial class	Number of isolates (%)		p-value

		Swine (n = 19)	Poultry (n = 23)	
Non-MDR				-
TET, P (G)	2	0 (0.00)	2 (8.70)	
TET, P (G), AML, and AMP	2	9 (47.37)	0 (0.00)	
Total		9 (47.37)	2 (8.70)	
MDR				0.001
TET, P (G), and SXT	3	0 (0.00)	5 (21.73)	
TET, P (G), AML, AMP, and SXT	3	8 (42.10)	1 (4.35)	
TET, P (G), AML, SXT, and CIP	4	0 (0.00)	2 (8.70)	
TET, P (G), AML, AMP, SXT, and CIP	4	2 (10.53)	5 (21.73)	
TET, P (G), AML, AMP, SXT, CIP, and NOR	4	0 (0.00)	6 (26.09)	
TET, P (G), AML, AMP, SXT, CIP, NOR, and AMC	4	0 (0.00)	1 (4.35)	
TET, P (G), AML, AMP, SXT, CIP, NOR, and GEN	5	0 (0.00)	1 (4.35)	
Total		10 (52.63)	21 (91.30)	

P (G)=Penicillin G, AML=Amoxicillin, AMP=Ampicillin, AMC=Amoxicillin-clavulanate acid, CIP=Ciprofloxacin, NOR=Norfloxacin, TET=Tetracycline, GEN=Gentamicin, SXT=Sulfamethoxazole-trimethoprim, MDR=Multidrug resistance

## DISCUSSION

The study findings indicated a low prevalence of *Salmonella* spp. among swine and poultry with diarrhea on small- and medium-scale swine and poultry farms in Cambodia, with an overall prevalence of 6.58%. Specifically, the prevalence of this disease in swine was 6.96%, which is notably lower than that reported in other countries. For instance, recent studies by Tadee *et al*. [15], Anuchatkitcharoen *et al*. [19] and Prasertsee *et al*. [20] in Thailand have documented prevalence rates ranging from 21.51% to 69%, whereas in Vietnam, reported prevalence varies between 14.58% and 87% [16–18]. The differences in prevalence could be explained by differences in farm management strategies, sanitation, hygiene practices, and the individual environmental conditions of each farm [30], and maybe a production type, farm scale, and targeted sample collection (e.g., healthy or sick animals). Most of the isolates in this study were collected from diarrhea feces of swine that were reared on medium-scale farms and may have sufficient biosecurity measures. This may have contributed to the lower prevalence observed in this study. In this study, the prevalence of *Salmonella* in growing swine was significantly higher than that in weaning and finishing swine (p < 0.001). This difference may be attributed to factors such as diet composition and feeding practices, farm personnel’s biosecurity measures, and the farm environment. Stress due to reduced feed availability has been identified as a contributing factor to the increased proliferation of microorganisms and swine infections, alongside bacterial load and individual animal health status [31]. In addition, poor biosecurity practices among farm workers, owners, and veterinarians may facilitate the direct or indirect transmission of bacteria to pigs and their farm environment. In Cambodia, a study by Chea *et al*. [32] reported that several biosecurity guidelines are not well understood or consistently implemented by swine farmers and village animal health workers. Furthermore, the farm environment serves as a potential reservoir for *Salmonella* transmission within and between herds, as well as across farms and regions. A study conducted in Vietnam found that *Salmonella* was present in the surroundings of 6.40% of swine farms [18].

The prevalence of *Salmonella* in poultry was 6.30%. Similar studies have been reported in broiler farms in Vietnam (8.24%) [33], Nepal (10.6%) [34], Nigeria (15.9%) [35], and China (11.2%) [36]. The environment, pest animals, and personal hygiene of farmers may contribute to the prevalence of *Salmonella* in poultry. According to our observations, most poultry farms are open-house systems, and there were not enough biosecurity precautions in place to prevent access by humans, rodents, wild birds, and insects. The presence of poultry buyers, neighbors, workers, and veterinarians on poultry farms increases the risk of disease introduction if biosecurity is not practiced [37, 38]. These individuals are considered high-risk groups for bringing diseases to poultry farms. Due to their regular and frequent presence on farms, farm workers, owners, and veterinarians, in particular, may help spread the bacteria to the poultry and its surroundings. *Salmonella* can infect poultry on a farm by residing in areas such as the barn floor and in the bedding, feed, water, and waste water. A recent study by Nguyen *et al*. [33] in Vietnam reported that 4.33% of poultry environmental samples were positive for *Salmonella*, which comprises bedding (5.88%), feed (5.48%), and drinking water (0.70%). The presence of pest animals on poultry farms could result in the transmission of *Salmonella* to poultry. The same study in Vietnam found that the prevalence of *Salmonella* was highest in rats (15.63%) and geckos (12.25%), followed by ants (2.83%) and cockroaches (2.44%) in poultry farms [33].

In the livestock farming especially swine and poultry production, antimicrobials are extensively used for therapy, prophylaxis, metaphylaxis, and growth promotion [12]. The most commonly used antimicrobials are β-lactams, tetracyclines, sulfonamides, aminoglycosides, and fluoroquinolones. Thus, this extensive and inappropriate use of antimicrobials has contributed to a genetic bottleneck that promotes the spread and dissemination of resistant genotypes among enteric bacteria, including *Salmonella* [30]. *Salmonella* isolated from swine and poultry in this study exhibited a high degree of resistance to tetracyclines (TET), sulfonamides (SXT), and β-lactams (PG, AML, and AMP), ranging from 73.81% to 100% of isolates tested. As shown in Table 2, they exhibited low resistance to the aminoglycoside group (GEN) (2.38%) and medium resistance to the fluoroquinolone group (CIP and NOR) (29.77%). The study conducted in China also found that large-scale intensive poultry farms had low resistance to GEN (14.90%) but high resistance to AMP (68.7%) [36]. Additional recent studies by Nguyen *et al*. [33] conducted in Vietnam have shown that *Salmonella* in poultry and in the farm environment is frequently resistant to TET (55.80%), AMP (54.14%), and SXT (53.04%). Similarly, in swine and their farm environment, *Salmonella* are frequently resistant to trimethoprim/sulfamethoxazole (64.47%) and AMP (46.05%) [18]. These two studies in Vietnam also found low resistance to the fluoroquinolones group (levofloxacin and ofloxacin) (6.08%) and (1.98%) in poultry and its farm environment and in swine and its farm environment, respectively. Studies also found low resistance to the aminoglycoside group (GEN) (7.89%) in swine and its farm environment and in poultry and its farm environment (6.63%). Possible reasons for the high/low resistance could be the emergence of AMR strains, which have been associated with the consumption of antimicrobials in veterinary medicine and low awareness among the key stakeholders on AMU and resistance. AMU studies in the Southeast Asian region, mostly from Vietnam, indicated very high usage levels of most types of antimicrobials, including β-lactams, aminoglycosides, macrolides, and quinolones [39]. In Cambodia, β-lactams (AML and AMP), aminoglycosides (GEN), tetracyclines (oxytetracycline), macrolides (tylosin), quinolones (enrofloxacin), and polymyxins (colistin) have been commonly used in swine [24]. Low awareness of AMU and resistance led to the emergence of AMR. Some studies in the region have reported that swine and poultry farmers/producers had limited knowledge and practices regarding AMU and AMR in Vietnam [40] and Thailand [41]. In Cambodia, low awareness has been reported among swine and poultry farmers/producers [24, 26, 42] and among veterinary professionals and drug retailers [43, 44].

In this study, all *Salmonella* strains exhibited resistance to 2–5 antimicrobial agents, with eight distinct resistance patterns identified in poultry and 2–4 antimicrobials, with three resistance patterns observed in swine. Notably, the prevalence of MDR was significantly higher in *Salmonella* isolates from poultry (91.30%), which exhibited resistance to three to five antimicrobial classes, compared with those from swine (52.63%), which demonstrated resistance to three to four antimicrobial classes (p < 0.001). Similar findings have been reported in Vietnam [18, 33], where MDR in *Salmonella* isolates from swine and poultry ranged from 72.37% to 100% of the isolates tested. However, these results differ from a study conducted in China, which reported a lower prevalence (53.70%) of MDR in broiler isolates [36]. The high resistance and MDR levels observed in this study may be attributed to prolonged AMU, frequent administration of specific antimicrobials, and inappropriate use. Scur *et al*. [45] have reported that extended AMU plays a key role in the selection and persistence of AMR *Salmonella* strains.

Furthermore, resistance to frequently used antimicrobials varies according to geographical locations, production practices, and AMU patterns [46]. Non-rational AMU is associated with a higher prevalence of AMR bacteria. A study by Ström *et al*. [24] conducted in Cambodia reported an increased prevalence of AMR on swine farms that administered antimicrobials prophylactically and in those that routinely treated entire groups or herds during disease outbreaks. This may also happen at Cambodian poultry farms. Another study reported that swine and poultry producers commonly use antimicrobials as primary treatment for sick animals without a specific diagnosis. In addition, antimicrobials were administered to entire animal groups when only one or a few individuals exhibited illness and were frequently incorporated into water or feed [26].

MDR can be caused by the accumulation of natural and acquired resistance because of the acquisition or alteration of genes that regulate resistance. Therefore, bacteria can become susceptible to a few antimicrobials and resistant to several antimicrobial agents or groups [47]. Resistance to multiple antimicrobials makes the treatment of infections caused by pathogenic bacteria, including *Salmonella*, difficult not only in swine and poultry but also in humans. Thus, the use of antimicrobials on commercial swine and poultry farms should be controlled and monitored to prevent the occurrence of pathogenic strains resistant to multiple antimicrobials, especially *Salmonella*.

## CONCLUSION

This study provides essential insights into the prevalence and AMR profiles of *Salmonella* spp. isolated from small- and medium-scale swine and poultry farms in Cambodia. It is one of the first to systematically assess *Salmonella* prevalence and AMR patterns at the farm level in Cambodia, offering a novel perspective on AMR in livestock before slaughter. Unlike previous studies that primarily focused on post-slaughter contamination, this research provides critical baseline data on *Salmonella* distribution in live animal production settings. The findings offer new insights into MDR patterns and underscore the urgent need for AMR surveillance and antimicrobial stewardship programs in the Cambodian livestock sector. The overall prevalence of *Salmonella* was 6.58% (42/638), with 6.96% in swine and 6.30% in poultry. The isolates exhibited high resistance to β-lactams (PG, AML, and AMP), tetracylcines (TET), and sulfonamides (SXT), with resistance rates ranging from 73.81% to 100%. Moderate resistance to fluoroquinolones (CIP and NOR) (29.77%) and low resistance to aminoglycosides (GEN) (2.38%) were observed. MDR was detected in 91.30% of poultry isolates and 52.63% of swine isolates, with resistance spanning three to five antimicrobial classes. These findings highlight the potential risks associated with AMR transmission in the food chain and emphasize the necessity of immediate intervention strategies to control AMR in animal sector.

Despite its strengths, this study has certain limitations. The research was conducted in only five provinces, limiting its generalizability to the entire country. Sample collection was restricted to farms with animals exhibiting clinical signs of diarrhea, which may have influenced prevalence estimates. In addition, molecular characterization of resistance genes was not performed, preventing a deeper understanding of the genetic basis of AMR. The absence of longitudinal sampling further restricts the ability to assess temporal trends in AMR development.

Future research should expand surveillance to include a broader range of farm types and regions to enhance representativeness. Molecular analysis of resistance genes would provide more detailed insights into resistance mechanisms. Investigating potential environmental reservoirs of *Salmonella*, such as feed, water, and bedding, could help identify sources of contamination. Longitudinal studies are necessary to track AMR evolution over time and evaluate the effectiveness of intervention strategies. Furthermore, understanding the role of farm management practices in the dissemination of AMR *Salmonella* is crucial for developing targeted biosecurity guidelines. The study provides a foundation for future epidemiological research and policy development aimed at mitigating AMR risks in animal production and ensuring food safety in Cambodia.

## AUTHORS’ CONTRIBUTIONS

KK: Conceptualization, supervision, and project management. BC: Conceptualization, methodology, and drafted, reviewed, and edited the manuscript. NB: Sample collection and testing. ST and SK: Data review and validation. RC, VV, and CF: Co-supervision and reviewed the manuscript. All authors have read and approved the final manuscript.
